# One size (doesn’t) fit all: new metaphors for and practices of scaling from indigenous peoples of the Northwest Amazon

**DOI:** 10.3389/fpubh.2023.1166134

**Published:** 2023-06-28

**Authors:** Kurt Shaw, Rita de Cacia Oenning da Silva

**Affiliations:** Usina da Imaginação, Florianópolis, Brazil

**Keywords:** early childhood, Amazon (Brazil), indigenous knowledge, scaling, anthropology

## Abstract

Ten years of field research and collaborative development of programs for early childhood in the Upper Rio Negro region of the Amazon provide the authors with new metaphors for achieving wider social impact and new frames to add to the international debate on ‘scaling’ social change initiatives. Using anthropology and ethno-ontology to think questions of universal and particular, center and periphery, the article reflects on the dangers of monolithic scaling to cultural diversity and future innovation. Instead of the metaphor of scaling — adopted in the discourse of public policy and international development from the Fordist or Taylorist efficiency of the economy of scale — indigenous people speak of exchange, sharing, and transformation. These ideas seek to connect local and decolonized models and value the diversity of local knowledges, epistemologies, and practices around early childhood development. Based on the expansion of the CanalCanoa project among diverse indigenous communities, the paper proposes a flexible and bottom-up model of achieving impact at scale through empowering local actors to teach each other and establish local criteria of learning and evaluation.

## 1. Introduction

One evening in 2018, after a group of Baniwa and Coripaco families came together to see a film on early childhood development made in collaboration with local indigenous experts, an older women shared an insight she had been developing for some time. “For some kinds of illness, we know that our traditional ways are the best. When a child is sick from *quebranto* or *pitiú* [forms of diarrhea and illness caused by the evil eye], I know that the hospital cannot help me, but a shaman can. On the other hand, I see that the white way to cure a broken femur works better than the way we cured that for many years; I will go to the hospital if my grandson breaks a leg. The challenge is for those things that come in the middle: how do I decide what works?”

The discussion that followed did not offer any simple answer to the Baniwa grandmother’s question. Instead, the group concluded that everyone in the community should keep talking and evaluating, trying to understand each case, learning what worked, and evaluating based on locally valid criteria. This paper proposes a similar response when considering impact at scale. Certain kinds of interventions lend themselves to a what we will argue is a classically European form of scaling, where one tries to find an ideal intervention and then adapt it for the complexities of diverse contexts. Other kinds of impact demand a different kind of learning and creativity, more similar to what we will describe as an Amazonian methodology of impact at scale. As the Baniwa women eating the fish and pepper stew in concluded, the hardest cases lie in the middle, demanding thought, research, discussion, and creativity. We hope this paper provides insights into how to think a larger range of possibilities for impact at scale, as well as providing insights into the relations among media, agency, and voice in the development of anthropological theory and public policy.

Over the course of almost a decade, Usina da Imaginação, with the CanalCanoa project, collaborated with indigenous leaders, midwives, shamans, educators, and public policy professionals to script and then film traditional child-rearing practices of indigenous groups on the upper Rio Negro. Indigenous intellectuals then showed the seven resulting films on early childhood to dozens of neighborhood groups in small cities, using the films as a stepping stone for conversations about how urban-indigenous families could adapt traditional best practices to a new and modern context.^1^ After the success of these “*ajuris de conhecimento*” (literally “knowledge barn-raising sessions”) — where local families dramatically increased multilingual education, improved nutrition for their children, and strengthened local support networks for young children (1) — we have observed and accompanied an expansion of impact at scale around the whole region, a process where many people and groups share agency, and with very little intervention from funders or the government. The results of this de-centered, rhizomatic, and often chaotic process provide important new frames and concepts — often emerging from indigenous Amazonian philosophy — that can contribute insights to the international debate on “scaling” social change initiatives.

The structure of knowledge development and reproduction among indigenous people in the Amazon does not fit the metaphor of scaling used by most international funders and government agencies. Ideas of scaling emerged largely during the second industrial revolution, when Fredrick Taylor’s “scientific management” and Henry Ford’s production line showed that large scale enterprises could produce goods more cheaply and efficiently. Many other fields then generalized this “economy of scale” to other fields, including government and social services.

The immense diversity of cultures — each with specific and often conflicting rituals, beliefs, and practices around pregnancy, childbirth, and early childhood — makes it impossible to create any single protocol for maternal and child health, caregiver education, or nutrition, the first element for producing a scalable “product.” In this paper, we think together with indigenous people to find other metaphors for impact at scale; these metaphors can help us to understand better ways to work with indigenous and other minority groups, but they also develop insights into how successful models can better promote local autonomy and creativity while reaching large numbers of people.

This paper follows a three step argument:

1. An explanation of the CanalCanoa methodology and how it emerged both from systems of indigenous knowledge exchange and dialogic education.
2. An analysis of nine different forms of replication, inspiration, and transformation of the CanalCanoa model in the upper Rio Negro region, focussing on how local indigenous communities used the work to become social agents around the public health of young children.
3. Using the epistemology and education of the Amazon — how indigenous people come to know their complex world and then intervene in it — we show how these forms of adopting and adapting knowledge and practice provide important lessons to impact at scale in any context.

This reflection opens new ways of thinking the connection between world-view and impact at scale, so that funders and practitioners can propose and evaluate other forms of scaling in non-European contexts.

## 2. CanalCanoa

Given that different communities face different challenges around how to raise children as they come to face technology, schools, and the national economy, CanalCanoa emerged as a way for various ethnic groups to dialogue, think, and strategize early childhood education as indigenous families move from traditional villages to urban centers. CanalCanoa began by developing films that documented and explained traditional child-rearing practices: indigenous leaders, children, midwives, shamans, grandparents, parents and other community members suggested both content and structure for the script, made the arguments, and provided interviews and visual examples. This was no easy task, given the extraordinary geographic, linguistic, and cultural diversity of the region. Remote towns like São Gabriel da Cachoeira are accessible only by plane or boat, and reaching many indigenous villages requires a 15-day canoe trip from there. There is also a wealth of linguistic diversity in the region, with 22 languages spoken among 27 ethnic groups.^2^

With drafts of the films in hand, indigenous educators then facilitated community gatherings called *ajuris de conhecimento: ajuri* means “collective effort” in Nheengatu, so the full meaning would be similar to “knowledge barn-raising sessions.” In traditional villages, the whole community comes together early every morning to discuss concerns of the day and develop a response. The *ajuris —* as a place where parents and grandparents could join neighbors to brain-storm the challenging of rearing a child in the city — served a similar purpose. The indigenous educators organized the ajuris in several radically different contexts:

1. In urban indigenous communities, where families from the villages have migrated in search of work and education, often leading to a crisis in indigenous identity, language, and practice.
2. In villages close to town, where indigenous migrants from distant villages try to maintain traditional forms of living while having easier access to banks, markets, and schools.
3. In distant villages (sometimes as far as 10 days away by canoe), where contact with modernity is more distant and irregular.

The films and *ajuris de conhecimento* were organized around seven themes that local communities identify as priorities for child development: (1) pregnancy and childbirth, (2) child stimulation, (3) language and song, (4) education, (5) protection and care, (6) orientation, and (7) public health. Each *ajuri* began with a short animated or fictional movie made by CanalCanoa on the basis of local stories and legends, with live acting or drawings made by indigenous children.^3^ After these playful shorts, the children moved off to play and adults would watch a 10 to 12-min film about one of the seven key themes. The indigenous educators then facilitated conversations with families and helped think through ways that the group could make a difference in the problems that concerned them. For the next seven weeks, the process would repeat on different themes. During *ajuris*, participants would share traditional meals from the region, including açaí, game, fish, and other items prepared using traditional methods, which are often unavailable or inaccessible in urban settings. The workshops followed the logic of traditional indigenous festivals, taking some elements from the *dabucuri* — a traditional ritual in the region that formalizes the exchange of goods and ideas with new visitors and other cultures (2) — while using aspects from other social encounters as well. The popular educators organized dinner for all participants — generally *mujeca* or *quinhampira* (peppery fish stews), açaí with manioc *farinha* or tapioca, or other local foods — to take advantage of the comensality that both anthropologists and local leaders cite as so important in the region (3).

The most important result, however, was not these pragmatic actions in themselves, but the improved social networks that emerged from them. As families move from villages to the city, they lose the strong intergenerational support networks necessary to raising children. By bringing parents and grandparents together – often with shamans, midwives, nurses, and public health agents – CanalCanoa re-created this informal social safety net. Every single interviewed participant mentioned strong social supports as a benefit of the project.

CanalCanoa showed the seven movies to over 50 groups of 10–15 parents from 24 indigenous communities, who viewed and discussed the films over the course of two months. 1,186 adults and 1,148 children participated in the project, and the evaluation conducted at the end of the *ajuris* showed improved results in 1) intellectual stimulation of babies and small children, 2) better nutrition and healthcare for families, and 3) stronger support networks(1). In interviews, 91% of participants reported that after the *ajuris* they spoke with their children and grandchildren more often in indigenous languages, told more stories, and sang more; in 56% of the urban *ajuris*, participants began to plant food at home. Three-fifths of participants said they had learned how to complement traditional and western medicine. In 70% of the *ajuris*, participants used more herbal and home remedies and in more than half of the groups, older women began to sell or distribute herbal remedies.

## 3. CanalCanoa: nine examples of impact at scale

The funding for CanalCanoa ended in 2019, but the authors of this article continued in contact with indigenous leaders, friends, and educators over subsequent years and returned in 2022. In spite of the COVID-19 pandemic and quarantine, which had terrible consequences for indigenous populations in the region, local people developed at least nine different projects we see as inspired, catalyzed, or motivated by CanalCanoa, and which we detail and examine in this portion of the paper. Most of these initiatives emerged from indigenous people with no or very little formal funding, and we see them as a powerful lens through which to see other ways that decentralized and decolonial impact can happen at scale.^4^ Most importantly, indigenous individuals and groups imagined, initiated, and brought these examples to life: though the ideas emerged in dialogue with CanalCanoa and the films it produced, the list that follows exemplifies cultural traditions of learning from and transforming what indigenous peoples learn from others.

### 3.1. Upriver

The Pastoral da Criança (Children’s Vicarate) is one of the most effective and wide-reaching programs for small children in Brazil. Run by the Catholic Church, the program trains community members (generally women seen as “successful” mothers or grandmothers) as educators for young parents, teaching about care during pregnancy and childbirth, nutrition for young children, and intellectual development in early childhood. The program is practical and pragmatic, and often gives substantial autonomy to the local educators [See (4, 5)].

Several Pastoral da Criança educators participated in the *ajuris* in São Gabriel and began to use CanalCanoa films and methodology in their work; this adoption led to a conversation where the Pastoral da Criança suggested replicating the CanalCanoa methodology in a dozen villages up many of the rivers in the region, some as many as 10 days by canoe from the urban center of the city. Over the next several month, CanaCanoa and Pastoral educators organized *ajuris* in more than a dozen different communities on four different river systems (see map), reaching close to a thousand people.


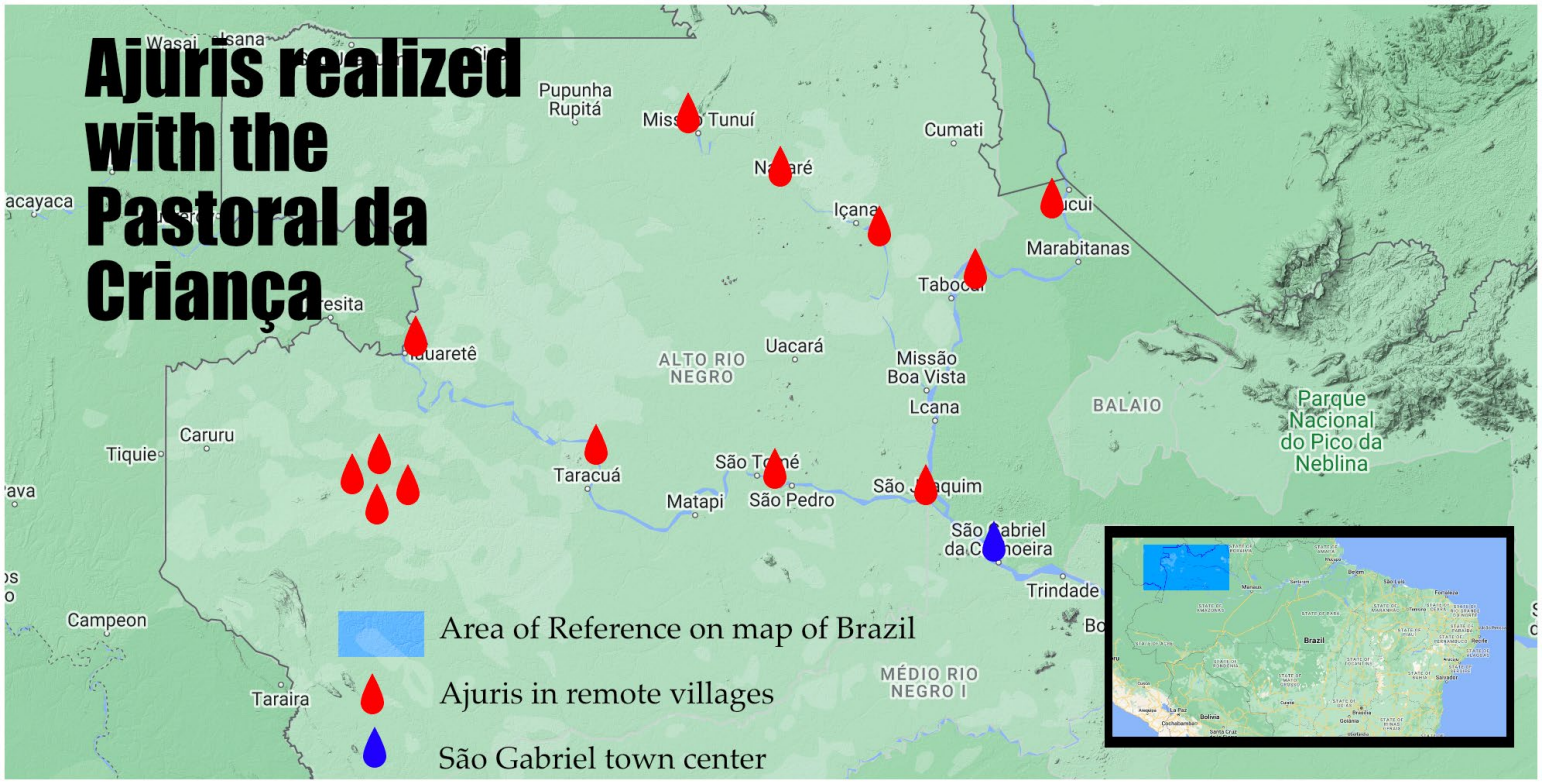


The extreme distances demanded certain changes: instead of one movie a week for seven weeks, the educators showed three to four films per day over an intense two evenings of movies and conversations. In the city, most *ajuris* were held in Portuguese, given that the groups were multi-ethnic and multi-lingual, but village sessions were spoken in Baniwa, Coripaco, Nheengatu, Tukano, Desana, and Tuyuka (fortunately, educators spoke all of these languages). Perhaps the most important difference, however, is that the conversations in the city largely revolved around how to re-constitute or adapt traditional child-rearing to a new world, while in the villages people used the films to understand how precious and unusual their traditions were, thus focussing on maintenance more than adaptation.

When asked to evaluate the *ajuris*, parents and grandparents in the villages emphasized the way that the discussions had helped them to evaluate and resist the pressures to move their families to the cities; in fact, the discussions had helped them to elaborate what an academic might call “evaluation tools,” which had previously been implicit in their thinking. Many families move to the city “for the good of our kids,” because only the urban area has a high school. Others go because of the challenge of receiving government benefits: welfare and social security checks must be cashed every three months, and when a trip to the bank demands ten days downriver and ten days back up, the temptation to stay in the city is powerful. For others — like for village dwellers everywhere in the world — the city seduces with an air of modernity, hope, and change. Participants in the *ajuris* did not say that the films and discussions motivated them to stay or go, but they said they provided new tools and insights to make their decisions and to take advantage of the best parts of the decisions they made.

Finally, these village *ajuris* were some of the most creative and productive in developing new forms of impact and scale. We address these developments below in 3.5–3.7.

### 3.2. Teaching indigenous pediatrics

The Tukano and Desana village of Balaio lies some eight hours from São Gabriel on an almost impassable road; during our 2019 trip to the village, the jeep drivers literally had to build their own bridges over 5 meter wide, 6 meter deep craters opened by seasonal rains. Everyone knew than the ancient village shaman did not have many years to live, and no member of the younger generation had yet memorized the long stories and blessings essential to local medical practice, based on a tripod structure of herbs, blessing, and narrative. CanalCanoa movies inspired the community to film dozens of hours of interviews with the shaman in order to preserve his knowledge, now indispensable after his passing in 2020.

During the 2020–2021 quarantine, the shaman’s daughter and granddaughters began a new plan for impact. They founded a school for indigenous medicine, especially medicine dedicated to pregnancy, childbirth, and early childhood. In the middle of the pandemic, the school took an online form, with lessons on different medicinal herbs and how to prepare them. The shaman’s daughter — now the chief healer of the region — showed how to prepare the medicine as her own daughter filmed on a cell phone. Another daughter and son then edited the film (a skill they had learned in CanalCanoa workshops) and distributed the videos by WhatsApp and social media.

By 2022, the school of indigenous medicine had inspired and hosted a shamanic medical conference funded by the indigenous health system of the Federal Government — including traditional healers from all over the region and policy makers. The community had re-built the village *maloca* (great-house) as a space for rituals, events, healing ceremonies, and rites of passage. A well-cut 2.5 km trail traversed the local biomes and soil types where different medicinal herbs grow; local children and visitors used the trail to learn about both biology and medicine. The lead healer had been invited to the city several times to lecture on traditional medicine, especially during the pandemic, when São Gabriel had one of the highest mortality rates in the world. During our last visit, the community was building a greenhouse for a medicinal garden and developing a label and brand for low-cost sales of traditional medicine.

### 3.3. School lunches

The urban ajuris attracted parents who worked in many different areas. During one of the events — directed largely to a Baré community living close to the city center — the head of the procurement office for the local school cafeterias and her two young children participated. After the film on nutrition, the group collectively lamented how their diverse and healthy diet in the villages had degraded into industrialized food in the city. Some women offered to teach others how to turn their back-yards into gardens; others invited new friends to join them in their farm plots outside the city. The procurement director got on her cell phone and began to research federal school lunch policies.

After discovering that federal rules favored local and organic producers, the procurement officer sought out local indigenous rural cooperatives, evaluated their capability to produce and deliver their goods, and taught them how to apply for government contracts. A group of urban Tuyukas who kept collective manioc fields and tropical orchards some five minutes by canoe from the city won the contract.

Today, children in São Gabriel get local produce in their school lunches and snacks. Instead of crackers or pasta shipped from Manaus made from wheat grown in Argentina — wheat cannot grow in the Amazon and has never been a part of the indigenous diet — children now eat *chibé* and other forms of manioc porridge, tapioca and *beiju* instead of bread; they drink açaí, tucumã, pupunha, cupuaçu, and other local fruits instead of soda pop or boxed grape juice. Unfortunately, school closures due to the pandemic made it difficult to measure the results on childhood health, but local nutritionists and indigenous leaders are optimistic [For a more detailed analysis, see (1)].

### 3.4. Training new indigenous health staff

The indigenous health service along the Rio Negro amalgamates many different government agencies: the National Health Service (SUS), the indigenous health service (DSEI), hostels for indigenous health (CASAI), the municipal health secretary, and the military hospital (the city is an important border garrison). Though some staff are local — an increasing number of indigenous youth have trained as nurses and doctors — many more come from distant parts of Brazil and even abroad and stay for only a year or two. Interviews with new staff exposed prejudices we had imagined eradicated half a century ago, including fear that white children would be abducted by indigenous people, terror of cannibalism, refusal to allow indigenous food in the hospital, and brutally high levels of obstetric violence.

After observing the CanalCanoa *ajuris*, the indigenous health service contracted one of the team’s indigenous educators as staff anthropologist and trainer. She used the films and the dialogic methodology in training sessions for health staff from almost all of the different agencies in the years leading up to the pandemic, when quarantine forced the end of in-person training (6).

### 3.5. Theater groups

The upriver *ajuris* inspired some of the most creative brain-storming to solve local problems; the next three examples of impact at scale may be considered second or even third level impact, because they emerged from the second-level scaling already carried out by the Pastoral da Criança.

The seven principle films of the CanalCanoa corpus inspired most of the scaling initiatives we detail here, but the more playful films also had an important impact among a younger audience. Among the 54 films distributed on CanalCanoa USB drives — a medium chosen because even in the most remote villages, someone has purchased a smart-TV and runs it on solar energy or a gas generator — almost two dozen have scripts based on traditional legends, folk tales, myths, or bed-time stories. In some cases, indigenous children act out these stories as if they were a play or fictional movie; in other cases children’s drawings serve as the basis for animated cartoons; and other movies simply film the best adult storytellers in the region.

From the beginning of the *ajuris*, we recognized a powerful and productive dynamic: the audience saw the films as incomplete. During conversations with indigenous educators, many people would say, “I know a midwife from a different tribe who has an interesting perspective on that … why don’t you interview her?” or, “you might talk with a shaman who blessed my son when he was sick from …” We always followed up on these ideas, which enriched the films immensely; we would often joke that each edition of the film was version 2.1 or 3.2, as in software development.

Young people in several Baniwa and Coripaco villages saw the fictional CanalCanoa films in the same light: not as a finished movie to be watched, but as the inspiration for dialogue and challenge. Sometimes children and teenagers would insist that they had heard the story of the origin of fire in a different way or that they knew other tales of the conflict between the jaguar and the tortoise.^5^ In other cases, they wanted to include different kinds of stories so that other kids could learn from them.

As a result of these movies, conversations, and debates, the young people in at least three different villages along the Aiari and Içana rivers created theater and film clubs where they develop scripts from traditional stories, assign roles, direct actors, and present their plays at schools and *malocas* (great-houses) in both Baniwa and Portuguese. Some of these groups also planned to film their plays and distribute the result through pen drives and WhatsApp, but their communities are so distant that recent news has been slow to get to us.

At first, this impact at scale might appear distant from the goals of public health, but we must remember two things. First, one of the three legs of the Rio Negro healing process is narrative. As a patient takes medicine, he or she must always listen to the correct story. Whether because of the documented power of the placebo or of “bedside manner,” indigenous people in the Rio Negro believe they will only be healed when the cure is accompanied by the story. Second, the process of documenting and performing stories serves as a motivator to keep children in the villages, with the better health and nutrition that implies. As such, theater groups can play a significant role in public health, and not only cultural persistence.

### 3.6. Lullaby research nucleus

Song is essential to Baniwa women: they sing to grow their peppers, the center of their culinary traditions and their medicine. They sing their blessings, which mark their rituals and their healing. And traditionally, they teach their children through song. After an *ajuri* relating to song and language acquisition in the ethnically mixed community of Yamado, many of the women compared their own childhoods to that of their babies. Their mothers and grandmothers had sung many lullabys to them, they remembered, but when their babies had a hard time sleeping now it seemed so much easier to play the radio or an mp3 on the cell phone. When given the opportunity to think about the change in their relationship to sleeping babies, they saw the loss of song as one of their reasons that their children developed more slowly than they had, and that the youngest generation spoke Portuguese instead of Baniwa.

As they sat down in the brain-storming session, the women developed two strategies. First, they would use their cell phones for something else: they would go to their grandmothers and aunts — many of them who still lived upriver in the more traditional villages — to record their traditional lullabys and children’s songs. They would bring all of the songs together and share them in the community, practicing them together. And finally, they would return to the tradition of singing their own children to sleep. They recognized that the consequences would include language and culture, but also in the relationship with their babies — essential to early childhood development in the eyes of neuroscientists and Baniwa midwives.

Second, the women in the village voted to turn off the community electrical generator every Monday night for an evening of “children’s stories and jokes” in the Baniwa language. The television, they argued, replaced the great-house and the fireside as the privileged space for storytelling — and with it, Portuguese replaced Baniwa. This change is immensely important for the multilingualism that is essential to the Rio Negro, where kinship rules demand that one must marry a person that speaks a different first language than her own. It is also important for the development of executive function and the prefrontal cortex (8).

For the indigenous people of the Upper Rio Negro, resources and ideas flow up and down the numerous rivers of the region. By recording lullabys upriver, the women in the research group not only came back downriver — in their village close to town — but gave in exchange the resource of recognition. When they asked the older women upstream for permission to record their songs, they also showed those women that their voices mattered, that the lullabys they sang to their grandchildren deserved to be preserved and that these older women has something immensely important to contribute to early childhood development.

### 3.7. Seed bank

In the large village of Taracuá — some 120 km by river upstream from the town of São Gabriel — the film about nutrition and health inspired a long debate among a group of Tukano, Desana, Tariana, and other women. Though many of them regularly traveled up and down the river to town, the movie showed them the challenges that many urban mothers faced to feed their children. The lack of *farinha* (a special manioc flour produced in the region) and the expense of açaí, bacaba, tucumã, and other fruits made them aware of how fortunate they were to be able to produce and consume healthy, traditional food. They were particularly concerned at news that the state agriculture department pressured farmers close to town to use exotic or genetically manipulated forms of the manioc root (9).

In order to preserve and value traditional food and agriculture, these women created their own seed bank of traditional foods. “Seed bank” may be a poor translation — though some tropical palms and fruits reproduce through seeding, women plant manioc from the branches cut from the plant when the root is planted; local potatoes and pineapple have their own forms of germination — but the basic concept is correct: to document, store, and archive all of the different species and variants of edible plants.

Because of Rio Negro kinship practices — which require marriage with a partner who speaks a different mother tongue and comes from a different village — and because of the *dabucuri* festivals — where communities exchange dance, stories, food, and seeds — women from all over the region live in Taracuá. Each had brought different seeds with her to her new home, as well as new ways of preparing the food. Many foods also have their own stories — myths of the origin of the plant that also explain its nutrition, preparation, and dangers — and women from different regions also found their their stories were different. They decided to record this diversity as well.

This seed bank provided even more diversity to the diet of adults and children, helping with trace nutrients needed for early childhood development and providing a more regular food supply in a region with little refrigeration. Perhaps more importantly, however, the women began to send seeds downriver to where the jungle had been degraded, allowing their family members to plant more traditional food and provide a healthier diet for urban children.

### 3.8. Training early childhood educators

Criança Feliz is Brazil’s federal program to support early childhood development. Adapted from the Pastoral da Criança methodology in the last years before the 2016 parliamentary *coup d’état*, it has nonetheless survived two subsequent regimes. Among other activities, the program trains local women as family peer educators, so that they can teach young mothers good practices for nutrition and stimulation of babies and young children.

During the pandemic, the program hired a new coordinator for the upper Rio Negro; upon arrival, she discovered that the training materials provided to her were inadequate for the indigenous context where she was to work. She found CanalCanoa films and materials to provide exactly the tools she needed for training and discussion. First, she used the films and the *ajuri* methodology for her research, creating several focus groups of 10 young indigenous mothers. She then brought together her research with CanalCanoa publications to present a proposal for adaptation to the state-wide planning committee for Criança Feliz.

As a result of the conversations that emerged from these adaptations of the *ajuri* methodology, the local office of Criança Feliz began to integrate young indigenous mothers in city planning strategies. Mothers and grandmothers began to demand shaded benches for nursing, places to change their babies diapers, and green spaces for children to play. Though these demands are only now working their way through the city planning system, the support of Criança Feliz offers hope for real architectural change.

After the films, the young mothers also asked Criança Feliz to facilitate contacts with midwives and shamans; they reported that the migration to the city cut them off from the traditional medical experts who are so important in the village. As a consequence, Criança Feliz workshops now include time with local experts who live in the city, and Criança Feliz volunteers are trained to connect local women to traditional medical services as well as to hospitals and government health posts. Finally, Criança Feliz has developed a new sector just to deal with herbal remedies.

### 3.9. Post-modern shamanism

During an *ajuri* with a group of about twenty urban families close to the center of São Gabriel, one of the participants was impressed by the film called “Protection,” which included techniques on how to keep kids safe from dangers of life in the jungle, both natural (animals, disease, drowning) and supernatural (spirits of the forest, witchcraft, the evil eye). Indigenous and scientific forms of research both show that shamanistic cures work for many of these ills — whether because of the placebo effect, improved immune system response, herbal medicine, or some other explanation — but the participant in the *ajuri* lamented that few young people were learning these traditional cures “Kids like movies like this,” he continued. “If I could make a movie, maybe I could interest them. Pity I can’t.”

“Maybe you don’t know how to make movies,” another participant chimed in. “But you do have this thing.” She showed him her cell phone. “Record the lessons and the blessings on the phone. Send them by WhatsApp. People can play them, repeat them … what a great way to learn!”^6^

Over the next several months, the older shaman did exactly that, circulating lessons and recording blessing over WhatsApp. Many young men and some young women began to train with this resource, and both shamans and the CanalCanoa team were optimistic about the technique as a democratic way to have an impact at scale.

Three years later, follow up research discovered complications. The shaman related that he had been forced to end the experiment for a reason that neither he nor we had expected. Because WhatsApp messages can be copied and distributed, he could not control who received the lessons; he had come to learn that some of the recipients used his lessons for nefarious purposes. “Your medicine can be a cure or a poison,” he told us.^7^ “Ours as well. When I found that some young men were using my lessons for witchcraft, I stopped.”^8^ Here, we see how the process developed an evaluation criterion which many larger programs might not have considered or in a formal proposal: moral evaluation.

The shaman did not allow this negative result to stop what had generally been an effective experiment. Now he uses WhatsApp and other social media only as a way to attract students; he then teaches them in person, only after he has come to trust in their motivations for learning. The attention that the WhatsApp experiment brought to his work also earned him employment at the local hospital: CanalCanoa materials, pressure from the local indigenous federation (FOIRN), and grass roots demands forced the hospital to hire shamans and midwives to work alongside university-trained medical staff.

## 4. Lessons for impact at scale

“A scientist might look at a certain type of manioc and see how it grows better than anything else, so he says, ‘This is the perfect manioc. Everyone should use it everywhere.’ But he brings that type of manioc here where there is a different soil, and it does not thrive.” A Tukano shaman explained this idea to us before detailing the 19 types of manioc planted in his community. “Here by the river, we have many kinds of soil, and we need many kinds of manioc. Each quality [type] grows better in a different kind of soil. And we use it for something different” (10).

After many encounters with researchers and government agricultural agents, the shaman had seen that European agriculture looks for the ideal variant of a crop, then transforms the land and ecosystem with pesticides and fertilizers to grow that platonic agricultural ideal. He would later point out that the schools and health centers built by the government had the same logic; a school building in an indigenous village looked little different from schools or clinics he had seen on a trip to Brasília.

As we reflect on the lessons from the success of CanalCanoa, we want to use the Tukano shaman’s analysis of crop adaptation to think about social interventions for early childhood. The metaphor provides an excellent way of seeing why many social programs fail in minority communities, but also how a re-framing of impact at scale opens new possibilities for impact and social change. Here, we want to look at three of the many reasons that this way of thinking impact at scale is so powerful:

1. Creative responses to diverse local challenges.
2. Stakeholder ownership/*protagonismo.*
3. Diversity and resilience.

### 4.1. Creative responses to diverse local challenges

Krapels et al. (11), coming from another vantage point, make a similar argument to that of the Tukano shaman.

“Scaling frequently diminishes or even eliminates the positive effects seen at small scale. With scale, the elements surrounding the delivery of an intervention frequently change. Even if the content or curriculum of an intervention remain the same, consistency in effectiveness cannot be assumed as key delivery mechanisms may need to alter. Adaptations may be required in light of new delivery agents (e.g., different teachers, health workers), different target groups (e.g., different children, parents), and differences in prevailing social and cultural norms and economic circumstances.”

The logic of the “economy of scale” can often imprison policy designers in a limited concept of impact at scale. The example of CanalCanoa shows that social change does not always work like industrial design, but can instead flow^9^ in unexpected (and unexpectedly productive) directions. No policy expert could have dreamed up the multiplicity of uses that local indigenous people found for the ideas, films, and methodology that emerged from CanalCanoa. As such, the experience provides useful tools to think new metaphors for scaling and to develop creativity and autonomy in public policy.

The Brazilian anthropologist Eduardo Viveiros de Castro has argued convincingly that where Europe developed ideas and virtues based on representation – and thus the correspondence theory of truth and the Platonic theory of forms – Amazonian indigenous peoples developed a philosophy of perspective and relationship. In the Amazonian ethical-ontological world, the most important commandment is to gain the ability to see through the eyes of the other:

[In European thought,] subjects, just as much as objects, are the result of a process of objectification: the subject constructs himself or recognizes himself in the object that he produces, and he comes to know himself “objectively” when he manages to see himself “from outside,” as a “that” … Amerindian Shamanism appears to be guided by the opposite ideal. To come to know [a thing] is to personify [it], to take on the point of view of that which you want to know … transforming a “something” into a “someone,” another subject or agent (12, p. 258).

This insight lies at the base of the most important contribution that indigenous people can provide to re-think impact at scale. While European policy design tends to search for ideal solutions that can then be replicated or adapted in many different contexts (emerging from a Platonic concept of form and existence), Amazonian perspectivism tries on the skin of the other like clothes, learns from it, and then invents something new based on the encounter of these two perspectives. We see this most clearly, perhaps, in the way indigenous people took on the role of film-makers and then adopted and adapted digital technology to their own ends: recording lullabys, teaching shamanism, turning myths into children’s plays filmed on cell phones. Just as the hunter learns from his experience in the skin of a jaguar but will never hunt as a jaguar, these local innovators experienced digital technology and then molded it to their needs. Perspectivist philosophy, with its experimental form of learning, hybridizes into an immense diversity of different solutions.

### 4.2. Stakeholder ownership/*protagonismo*

Each of the innovations we listed in part 3 emerged from the diverse communities of the upper Rio Negro, each of them responding to local problems and based on local strengths. In addition to the creativity this process implies, it also fits into one of the great virtues of Latin American social thought: *protagonismo*.

Many Latin American social movements insist that when the *victims* of centuries of slavery, colonization, and oppression became the *objects* of government interventions, they only exchanged one form of passivity for another. In this perspective, good policy and education must instead create the circumstances where people become the active *subjects* or *protagonists* of their own lives, not passive victims or objects. Though the translation is inadequate, this idea maps across European or North American analyzes of the locus of social agency or of stakeholder ownership.

For this reason, the creativity and problem-solving of these different forms of impact at scale go far beyond the impact on pregnant women and young children. When indigenous individuals, families, and collectives learned that they could solve problems around early childhood, they saw that they could also solve other problems. They did not need to wait for the agency of the other — the government, an NGO, or some other external actor — but could act on their own. This lesson revived and renewed the traditional indigenous values of autonomy and self-help, often weakened by the encounter with the west and the difficult adaptation to urban, modern life.

The evaluation of the results of the *ajuris* shows that this sense of community ownership of the project was one of the reasons it made such an impact (1). Many interviewed participants explained that they learned to value their own knowledge after sharing it with others. “I have always known what was right when it comes to my babies,” one Baniwa mother told us, “but I haven’t always done it. You come to the city and everybody does it a different way, and you come to suspect you were wrong, that you should do it like the white people do. But we watched the movies, we talked, and I remembered what is right and started to do it like I should.” Other mothers explained that when they began to teach others about indigenous ways of raising children, they had to live up to their own teaching, to “practice what they preach.”

Another participant — a mother of five children, an elementary school teacher and wife of a health agent on the Içana river — told us, “you come to the city and no one values your culture. Other things matter. But when other people start to value what we know — and it appears on that TV screen — then we see that it really is valuable.” A Baré mother from the Ilha do Açaí said something very similar: “It’s like a mirror. If people from outside value it, we learn to do so too.” When a community sees itself as both the messenger and the message, as the producer and receiver of knowledge, it increases the success of the intervention. In this way, the community and its members also see the opportunity to control their own story and the way they see themselves presented to other people. Though this presentation will continue to be contested through mainstream media presentations, it does provide a new tool for indigenous self presentation and self-examination.

This community buy-in (or stakeholder engagement or *protagonismo*), has huge implications for sustainability. Because of community commitment and creativity — because they believed in their own way of raising children and were inspired to create new ways to preserve those traditions — each village, tribe, and neighborhood continued the project on its own and in its own way. Pierre Bourdeiu points to *habitus* — the way that habit and social pressure interact with body memory and context to establish the ways that people behave and interact — as what sustains a practice over time (13). Indigenous cultures have a long *habitus* around pregnancy and early childhood, but modernity and urbanization challenged that culture and habit. In an important way, the *ajuris de conhecimento* served as a way that indigenous people could adapt their *habitus* to the modern world in a conscious way, both in family relations and in the creation of new forms of social impact.

These new habits require inputs in order to be sustainable: cultural pressures, the mirror of the media, the effort of many people in the community. Importantly, money is not the most important factor in this sustainability. With the exception of the upstream replications by the Pastoral da Criança (in which we invested less than US$5,000 to pay for gasoline for outboard motors), none of these interventions required a cent of additional fundraising. In the villages and communities — the theater groups, seed banks, lullaby research nucleus — they demanded no funding at all. When the impact happened through transformation of government programs, budgets were adjusted but never increased.

### 4.3. Diversity and resilience

Perhaps the greatest challenge for scaling any government program for children is that indigenous families are different from the model assumed by the programs. In many indigenous groups, the nuclear family is not the prime agent of socialization of children. In some cases, other children or adolescents care for and educate little kids. In others, grandparents, godparents, uncles or aunts play this role. The Kaduvéu love children, but they think that pregnancy and childbirth are beneath them, so many parents adopt children from other communities (14). Among the Laklãnõ, young parents will give their first child to the paternal grandparents or great-grandparents to be raised as their child – even putting grandparents’ names on the birth certificates as parents (15). Among many Jê cultures, babies receive the names of deceased family members; interactions with those children are based not on play, but on the respect given to those elders. Regardless of the details, in almost every indigenous group in Brazil, the whole community is responsible for raising every child.

During one of our meetings with indigenous leaders and intellectuals to design CanalCanoa, we tried to explain how important it was for funders to support initiatives that could be scaled. “Scaling,” we explained, “is when you take a certain model that works in one place, and you implement it in many other places so that it can help more people.”

“Oh, like colonialism, then?” one of them responded.

For contemporary indigenous people in the Amazon, colonialism is not simply the presence of a foreign people occupying their territory. More than anything else, it is the idea that there is only one correct way to live in the world: a single best way to educate children, certain goods that everyone must consume to be included, one single model of what it means to be human. This colonization happens through individual colonists, but even more through television, schools, social workers, the internet … all of the different ways that contemporary society has developed in order to teach people to be workers, citizens, and consumers.

In the same way that the Tukano shaman pointed out that a strain of manioc that grows well in one field will fail in another soil, a single model simply may not work in other contexts. The resiliency of many alternatives might be equally important. In the same way that genetic monotony leaves plants open to plague and pests — the Irish potato famine being the classic example — monochromatic interventions may collapse before unexpected social change or may simply not work in contexts of diversity. The diversity of responses increases the chances that many of the variations will thrive over time.

## 5. Conclusion

The Baniwa grandmother at the *ajuri* in the village of Yamado compared what indigenous medicine does well with what Western medicine does well. We might engage in a similar exercise with impact at scale, comparing the Amazonian way being inspired by new perspectives with the Platonic search for an ideal intervention that can be adapted across the board. The Platonic model of scaling works well for certain kinds of interventions; the Amazonian form works for others. Undoubtedly, other kinds of epistemologies that emerge from Africa, Asia, and Oceania will contribute even more ideas to impact at scale.

We understand why the idea of scaling that emerges from the encounter of Platonism and industrial design is seductive to governments and funders. It standardizes and appears to treat everyone equally. It is less expensive and easier to implement. And it makes intuitive sense to policy makers trained in the European intellectual tradition. However, when we see only this form of scaling, we close our eyes to the multiplicity of creativities that emerge from other kinds of learning and impact at scale. Foundations and governments select projects that will fit into the European paradigm, while leaving out projects that might be more creative, resilient, and effective but that are more difficult to measure or to scale in an industrial way.

The experience of CanalCanoa — like that of many other cases in this special issue of *Frontiers in Public Health* — argues that we need to expand the size of our intervention tool-box, not to exclude the model of scaling that emerges from European and North American modernity, but to include the myriad of other forms of impact at scale that emerge from other cultures, other epistemologies, and other ways of learning and acting in the world. If scaling is a priority in a contexts of diverse and rich cultures — as is the case in the northwestern Amazon — one should not scale any particular model. Instead, one scales the *concept* of diversity and multiplicity, providing the support so that local people have the opportunity to learn and teach as they make an impact in the lives of small children.

## Data availability statement

The original contributions presented in the study are included in the article/supplementary materials, further inquiries can be directed to the corresponding author.

## Ethics statement

The studies involving human participants were reviewed and approved by CONEP Comissão Nacional de Ética em Pesquisa. The patients/participants provided their written informed consent to participate in this study.

## Author contributions

All authors listed have made a substantial, direct, and intellectual contribution to the work and approved it for publication.

## Funding

Funding for the CanalCanoa project (Usina da imaginação), came from Grand Challenges Canada, the Fundação Maria Cecília Souto Vidigal, and the Bernard Van Leer Foundation. This paper was funded with additional support from Grand Challenges Canada, to whom the authors express our gratitude.

## Conflict of interest

The authors declare that the research was conducted in the absence of any commercial or financial relationships that could be construed as a potential conflict of interest.

## Publisher’s note

All claims expressed in this article are solely those of the authors and do not necessarily represent those of their affiliated organizations, or those of the publisher, the editors and the reviewers. Any product that may be evaluated in this article, or claim that may be made by its manufacturer, is not guaranteed or endorsed by the publisher.

## Footnotes

^1^All of these films are available online at the Usina da Imaginação channel on Vimeo. The videos on Public Policy (https://vimeo.com/357015971) and Intellectual Stimulation (https://vimeo.com/357007417) have English subtitles. The films on Song and Language (https://vimeo.com/357005471), Pregnancy and Childbirth (https://vimeo.com/357009483), Nutrition and Health (https://vimeo.com/357010969), Protection (https://vimeo.com/357014382), and Orientation (https://vimeo.com/357012591) are available in indigenous languages and Portuguese.

^2^The anthropological literature on the region is equally complex and varied, with hundreds of scholars specializing in the different ethic groups. For an overview of this literature, we suggest the excellent article “Rio Negro Ethnic Groups” at the Indigenous Peoples of Brazil website, hosted by the Instituto Socioambiental. https://pib.socioambiental.org/en/Povo:Etnias_do_Rio_Negro. The page (in English) includes an overview, links to pages on each ethnic group, and an exhaustive bibliography.

^3^There were almost 50 of these films; local children and adults most liked “Long ago, fire did not exist” (https://vimeo.com/324968243), “The jaguar and the tortoise” (https://vimeo.com/240032370), “The Party in the sky” (https://vimeo.com/usinadaimaginacao/festanoceu), and “How to make a top” (https://vimeo.com/205583833).

^4^Usina da Imaginação — the Brazilian non-governmental organization that created CanalCanoa — has continued and extended this dialogue with indigenous peoples on public policy for indigenous children with the annual conference MIMUS: Multiple Childhoods, Multiple Knowledges. https://mimus.usinadaimaginacao.org/.

^5^Interestingly, Claude Levi-Strauss, working in another part of the Amazonian cultural sphere, developed much of his structural anthropology on exactly these same differences between common tales told among different indigenous groups (7).

^6^It is notable that many younger indigenous people saw the cell phone as a tool to make films; the ajuris and the presence of a film crew among them inspired grass-roots film-making. Older participants thought much more of the aural possibilities pf the cell phone, perhaps because they had become used to the voice messages on WhatsApp (the preferred means of communication in the region) or because they had experience with tape recorders in the past. In both cases, the locally produced films helped to re-frame accessible technology as a tool for culture.

^7^In Greek, the word φάρμακον can mean medicine, poison, or scapegoat, and indigenous languages and the Portuguese spoken in the countryside maintain this ambiguity. For more reflection on the subject, see (10).

^8^Baniwa and Coripaco people believe firmly in the power of witchcraft, which can be a challenge in conversations with an outsider. Whether witchcraft is true or not, however, isn’t really the point. When people on the Rio Negro believe they are the victims of evil magic, they become sick unless they have some stronger form of medicine to protect them. Even if witchcraft is not true, the symptoms (and the cure) are visible and measurable.

^9^Living in a world of rivers, the metaphor of flow is, of course, important in Amazonian thought.
